# Observation of Molecular Complexes in Oligo-Phenylenevinylene (OPV) Organogels by Neutron Diffraction

**DOI:** 10.3390/gels11020137

**Published:** 2025-02-15

**Authors:** Jean-Michel Guenet, Ayyappanpillai Ajayaghosh, Vakayil K. Praveen

**Affiliations:** 1 Institut Charles Sadron, CNRS-Université de Strasbourg, 23 Rue du Loess, BP 84047, 67084 Strasbourg Cedex, France; 2 Department of Chemistry, SRM Institute of Science and Technology, Chennai 603203, India; ajayagha@srmist.edu.in; 3 CSIR-National Institute for Interdisciplinary Science and Technology (CSIR-NIIST), Thiruvananthapuram 695019, India; vkpraveen@niist.res.in

**Keywords:** organogels, molecular structure, molecular complex, molecular compound, neutron diffraction

## Abstract

In an earlier report, we conjectured that oligo-phenylenevinylene (OPV) molecules bearing terminal OH groups may form molecular complexes in organogels prepared in benzyl alcohol. This assumption was based on circumstantial evidence only. In this paper, we report on new experimental evidence by means of neutron diffraction that unambiguously demonstrates this conjecture. After ascertaining that the thermodynamic properties of OPV gels are not altered by the use of a solvent isotope (hydrogenous vs. deuterated benzyl alcohol), we show that the neutron diffraction pattern in hydrogenous benzyl alcohol differs from that in deuterated benzyl alcohol. These patterns also exhibit additional peaks with respect to those obtained by X-ray. Comparison is further achieved with an OPV molecule without hydrogen bond terminal groups. In the latter case, no molecular complex is formed. These molecular structures may have a direct bearing on the differences observed in the gel morphologies.

## 1. Introduction

Organogels are fascinating systems as they form three-dimensional networks from solutions containing a low percentage of relatively small-sized molecules designated as organogelators or low-molecular-weight gelators (LMWGs) [1,2,3,4,5,6,7,8,9,10,11,12,13,14,15,16]. In a recent monograph, these molecules were regarded as “chimeras” due to the fact that they simultaneously bear different kinds of interactions, such as hydrogen bonding, π-π stacking, van der Waals, and the like [5]. Due to this unusual chemical architecture, their crystallization habit favours one specific growth face, which results in the creation of very long fibrillar entities that are more or less connected so as to produce an infinite network.

The knowledge of their molecular structure is a topical issue [5,6,7,8]. One specific point raised quite recently concerns the formation of *molecular compounds* from the co-crystallization of the organogelator and the solvent [5,6]. The expression *Molecular compound* is the generic terminology for systems that combine two or more molecules under a well-defined stoichiometry, independently of the way they interact. This term is customary in the nomenclature used in temperature–concentration phase diagrams [17]. In practice, several names can designate these systems depending on the type of interactions: *crystallo-solvates*, *molecular complexes*, *inclusion compounds*, *intercalates*, *guest–host compounds*, and the like [17,18,19]. Systems formed through hydrogen bonds are designated as *molecular complexes*, a situation that will be encountered in this paper.

Several authors have already suspected from circumstantial evidence that the solvent is not only a simple diluent but plays a role in the gelation process [20,21,22,23,24]. The existence of molecular compounds has only been recently demonstrated by means of neutron diffraction while investigating the gelation of triaryl trisamide molecules with a series of organic solvents [24,25]. It has been further shown that the occurrence of molecular compounds has a direct bearing on the gel properties [26,27]. Neutron diffraction, by making use of the isotopic contrast brought about by using either hydrogenous or deuterated species, can unambiguously demonstrate the occurrence of molecular compounds [25].

**Scheme 1:**
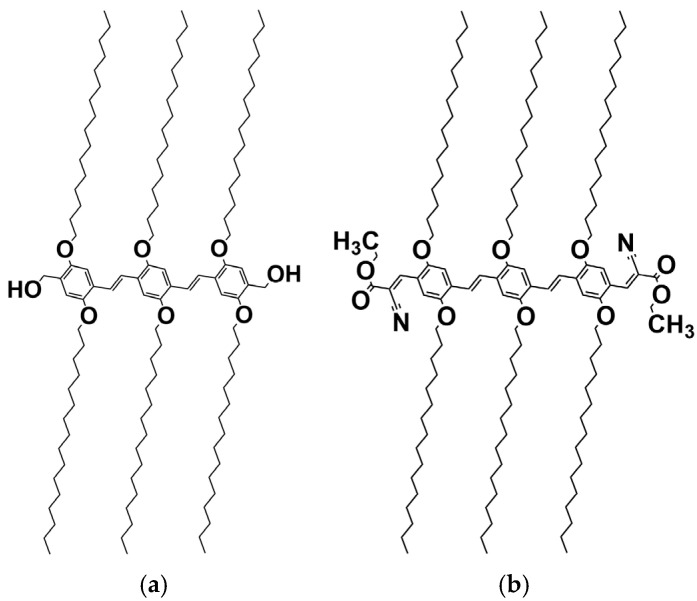
(**a**) Chemical structure in an all-extended conformation of OPVOH and (**b**) OPVR. The only difference lies in their terminal groups [28].

Based on circumstantial evidence, in previous papers, we have already conjectured that the occurrence of molecular complexes through hydrogen bonds in some OPVs should be contemplated [21]. Here, we present neutron diffraction data that definitely establish the existence of these complexes with benzyl alcohol and an OPV molecule bearing OH terminal groups (see Scheme 1). Conversely, we further show that no molecular complex occurs when the terminal group is replaced by a non-hydrogen-forming group [28].

Note that these systems were originally selected in previous papers for their unusual opto-electronic properties [21,28]. In particular, OPVOH gels change colour from yellow to green on melting, while OPVR gels convert from red to orange on melting. The colour depends also on the solvent type.

## 2. Results and Discussion

The oligo vinylene phenylene molecules investigated herein presented in Scheme 1 are typical “chimera” molecules [5,28]. They consist of a central core made of phenyl groups linked by ethylene groups, and six aliphatic arms containing 16 carbons each. The structure is either completed with two CH_3_-OH terminal groups that give a hydrogen-bonding character to this molecule (OPVOH Scheme 1a) and that mimic part of the benzyl alcohol or a NCOOC_2_H_5_ group (OPVR Scheme 1b) free of hydrogen-bonding character [28].

The present study of the molecular structure has been carried out by X-ray and neutron diffraction in systems prepared from hydrogenous benzyl alcohol (C_7_H_9_OH, called BzOH_H_) and deuterated benzyl alcohol (C_7_D_9_OH, called BzOH_D_).

### 2.1. Thermodynamics

In a previous paper, we reported temperature–concentration phase diagrams for OPVOH/benzyl alcohol and OPVOH/benzyl methyl ether [21]. The chemical structure of benzyl methyl ether (BME) is very close to that of benzyl alcohol, except that the OH function is replaced by OCH_3_, which impairs the formation of hydrogen bonds.

**Figure 1:**
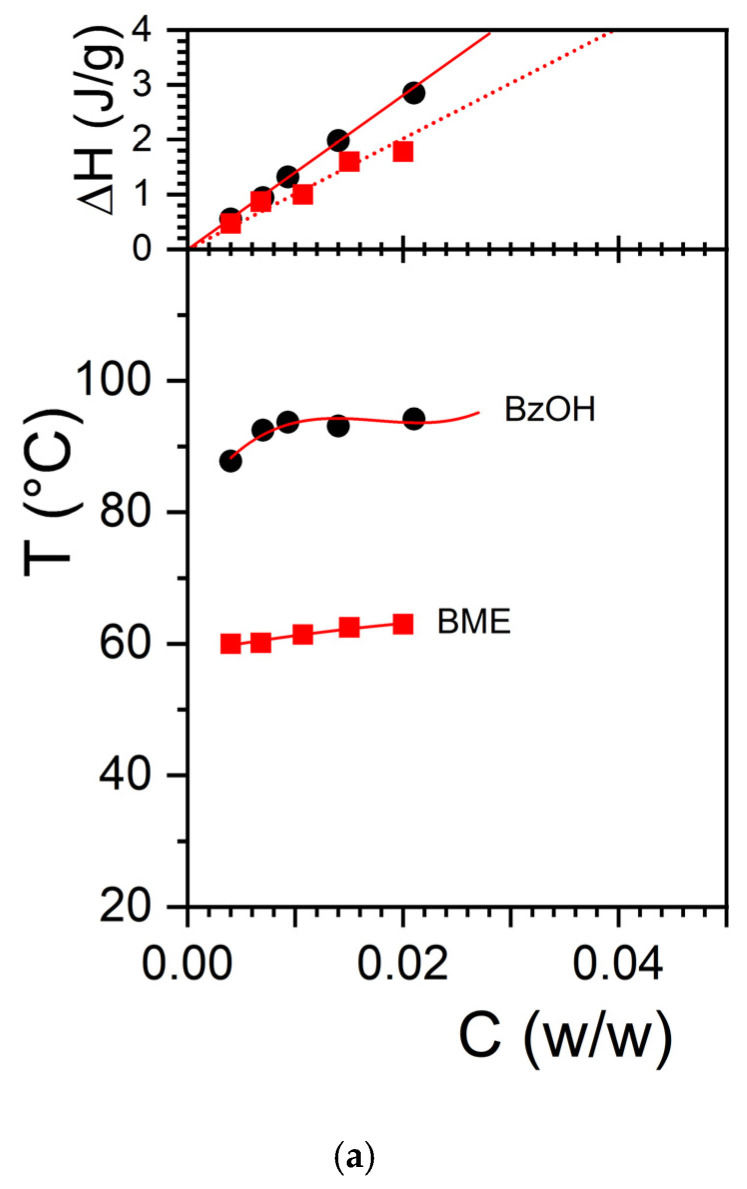

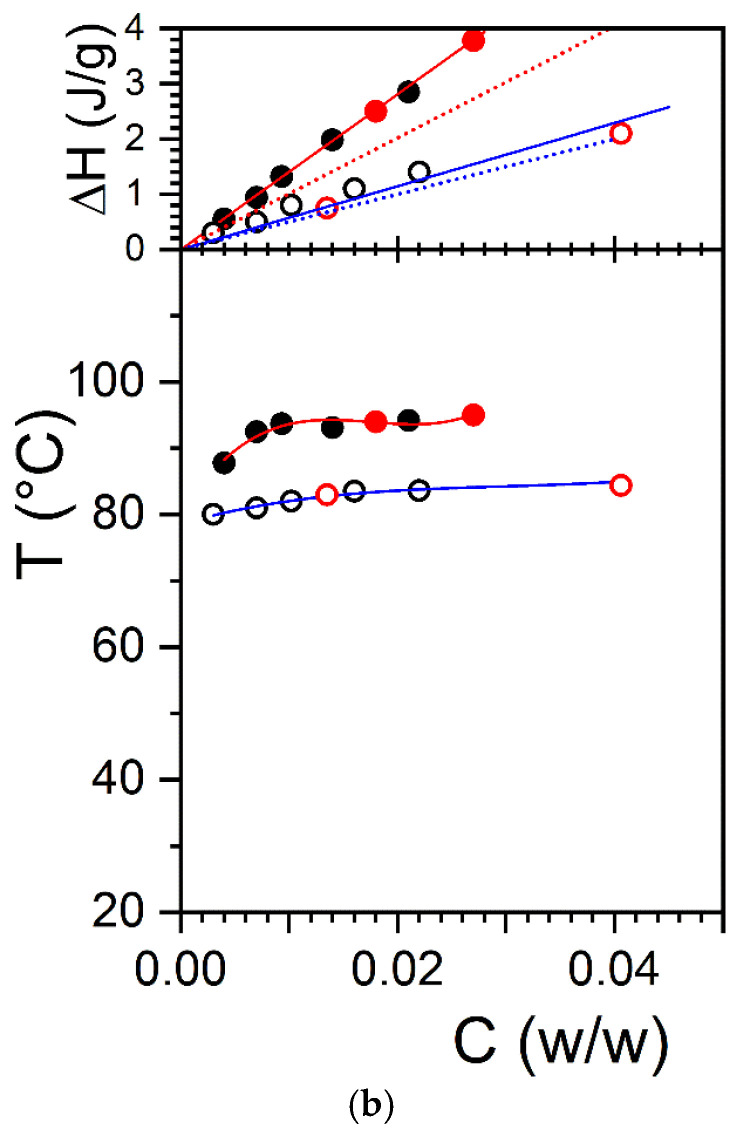
Temperature–concentration phase diagram (**bottom**) and Tamman’s diagram (**top**) for (**a**) the melting of OPVOH/BzOH organogels (●), and OPVOH/BME organogels (■); (**b**) the melting of OPVOH/BzOH_H_ organogels (●), OPVOH/BzOH_D_ organogels (●), OPVR/BzOH_H_ organogel (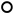), and OPVR/ BzOH_D_ organogels (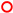). For the T-C diagrams, lines are guides for the eyes. For Tamman’s diagram, the solid lines are linear fits going through the origin (Gibbs phase rules), while the dotted lines represent the hypothetical behaviour if the melting enthalpy were the same as that in the solid state (ΔH_OPVOH_ = 181 kJ/mol and ΔH_OPVR_ = 98.6 kJ/mol, [21]).

The large difference in melting temperature between OPVOH/BzOH_H_ and OPVOH/BME together with the difference in melting/formation enthalpies led us to suspect that benzyl alcohol may form a molecular complex with OPVOH through the formation of hydrogen bonds (see Figure 1a). That the extrapolated melting enthalpy to C_OPVOH_ = 1 gives a value much larger than that measured in the solid state for OPVOH/BzOH gels as opposed to what is seen for OPVOH/BME supports the hypothesis of a differing molecular structure [21]. This is further observed for the OPVR/BzOH systems in Figure 1b, where the extrapolated enthalpy is found to be virtually identical to that in the solid state.

Yet, no direct demonstration has enabled us to confirm this conjecture so far. Note that this effect cannot be in any case related to the solvent quality, which does not affect the value of the melting enthalpy.

The T-C phase diagram of OPVOH and OPVR in hydrogenous and deuterated benzyl alcohol is shown in Figure 1. The use of deuterated benzyl alcohol instead of the hydrogenous version does not alter the thermodynamic properties of the organogels. The formation and melting temperatures are identical, as are the associated enthalpies. Comparing the neutron diffraction patterns obtained with hydrogenous benzyl alcohol and deuterated benzyl alcohol is therefore a legitimate procedure, something already well documented [29].

To extend this study further, we have studied another OPV molecule, namely OPVR, where the chemical structure of the terminal group theoretically prevents the molecule from establishing hydrogen bonds with benzyl alcohol (Scheme 1b). As can be seen, the melting temperature of OPVR in benzyl alcohol is lower than that of OPVOH. Unlike OPVOH, extrapolation of the melting enthalpy to C = 1 does give a value nearly identical to that measured in the solid state (Figure 1). In this system, the formation of an OPVR/benzyl alcohol complex is therefore not expected. Here too, there is no difference in the melting properties of OPVR organogels, no matter whether hydrogenous or deuterated benzyl alcohol is used (Figure 1).

### 2.2. Morphology

**Figure 2:**
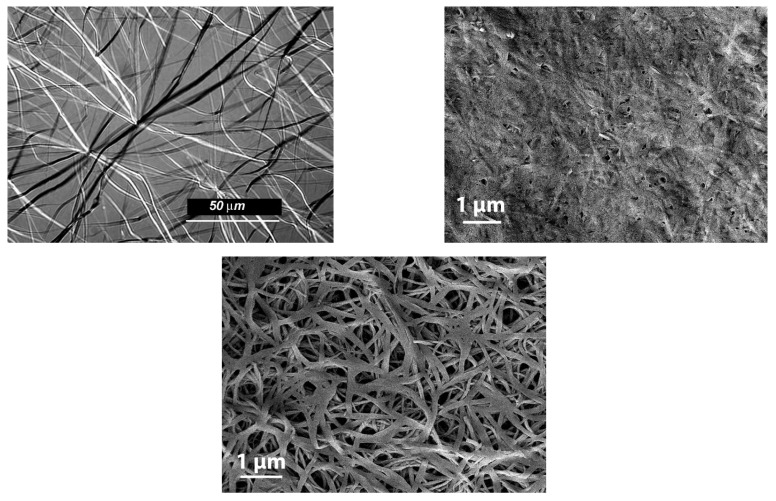
(**Top left**) optical micrograph of OPVOH/benzyl alcohol organogel. (**Top right**) FESEM image of vacuum-dried OPVOH/benzyl alcohol; (**bottom**) FESEM micrograph of vacuum-dried OPVR/benzyl alcohol organogel.

The morphology of these organogels is also strongly dependent upon the terminal groups.OPVOH/benzyl alohol have an unusual morphology of these gels, which is of the hub-like type with thin ribbon-like fibrils that radiate from well-identified centers (Figure 2 (top left)). Ribbons possess cross-sections of about 2 µm. Conversely, OPVR/benzyl alcohol organogels display a commonly observed morphology in organogels, namely randomly dispersed fibrils (Figure 2 (bottom)). Here, the cross-sections are smaller as they stand somewhere between 0.05 and 0.2 µm. Interestingly, drying OPVOH/benzyl alcohol gels to perform SEM investigations leads to an apparent collapse of the gel structure (Figure 2 (top right)) unlike what is seen with OPVR gels. This effect may arise from the dismantling of the OPVOH/benzyl alcohol molecular complex in the course of the drying process. This suggests that the simple OPVOH-OPVOH self-assembling process is not favoured.

### 2.3. Neutron Diffraction for Studying Molecular Compounds

The main technique for deriving the molecular structure is radiation diffraction, and more commonly X-ray diffraction. Neutron diffraction offers further possibilities, especially in the case of molecular complexes, thanks to the availability of isotopic labelling [30,31,32,33,34,35].

In the case of neutron diffraction, the intensity is written as follows [31]:

(1) $$I\left(q\right)=\frac{1}{N^{2}}\sum_{i}\sum_{j}a_{i}a_{j}{exp}^{iqr_{ij}},$$

where *a_i_* and *a_j_* are the neutron-scattering lengths of species *i* and j, and *r_ij_* is the associated distance.

For systems comprising two types of molecules, 1 and 2, (1) is written as follows:

(2) $$I\left(q\right)=\frac{A_{1}^{2}}{N_{1}^{2}}\sum_{i}\sum_{j}{exp}^{iqr_{ij}}+\frac{A_{2}^{2}}{N_{2}^{2}}\sum_{k}\sum_{l}{exp}^{iqr_{kl}}+\frac{2A_{1}A_{2}}{N_{1}N_{2}}\sum_{m}\sum_{n\neq m}{exp}^{iqr_{mn}}$$

where *A*_1_ and *A*_2_ are the scattering amplitudes of molecules 1 and 2, which are calculated by summing the scattering lengths of their constituting atoms ($A=\sum_{s}a_{s}$) [30]. Indeed, unlike X-rays, these scattering amplitudes are *q*-independent as neutrons interact only with the nucleus [30,32], whose size is negligible with respect to their wavelength. It is understood that only the terms where *A_m_* ≠ *A_n_* are considered for the third term of Relation (2). Relation (2) can be rewritten as follows:

(3) $$I\left(q\right)=A_{1}^{2}S_{1}\left(q\right)+A_{2}^{2}S_{2}\left(q\right)+{2A}_{1}A_{2}S_{12}\left(q\right)$$

For systems composed of a crystallizable component C and a solvent S, two cases can be contemplated. On the one hand is the occurrence of a *molecular complex* where the co-crystallization of both species takes place. A complex is characterized by a stoichiometric composition C_γ_, corresponding to the number of solvent molecules S per crystallizable molecule C. On the other hand, a *solid solution* may form where the solvent is totally expelled from the crystal. Note that we use the term “*solid solution*” in a broad sense here, as it normally implies, as is the case in metal alloys, that the minor component may randomly occupy some positions of the crystalline lattice of the major component. If the solvent can be found in the amorphous domains, as is the case for semi-crystalline polymers, then the use of this term is legitimate [36]. Now, in the case of organogels, there may not be such domains, so the term *liquid–solid system* would be more appropriate.

In the case of a solid solution or liquid–solid system, the cross-term in Relation (3) vanishes so that

(4) $$I\left(q\right)=A_{C}^{2}S_{C}\left(q\right)+A_{S}^{2}S_{S}\left(q\right)$$

Under these conditions, the diffraction pattern *I*(*q*) of the crystals from molecule C does not depend on the value of the scattering amplitude of *A_S_* (no change in the peak intensity nor in peak number).

In the case of molecular complexes, the cross-term cannot be neglected any longer. Also, there are two types of solvent molecules if the concentration is below the stoichiometric composition: those remaining liquid and those participating in the co-crystals. The intensity is written as follows:

(5) $$I\left(q\right)=A_{C}^{2}S_{C}\left(q\right)+A_{S}^{2}S_{S}^{co}\left(q\right)+A_{S}^{2}S_{S}^{liq}\left(q\right)+{2A}_{C}A_{S}S_{CS}\left(q\right)$$

where $S_{S}^{co}\left(q\right)$ is the diffraction arising from the solvent molecules belonging to the molecular complex and thus being organized, while $S_{S}^{liq}\left(q\right)$ corresponds to the diffraction of solvent molecules in the liquid phase. As a result, the diffracted intensity from the molecular complex reads

(6) $$I_{comp}\left(q\right)=A_{C}^{2}S_{A}\left(q\right)+A_{S}^{2}S_{S}^{co}\left(q\right)+{2A}_{C}A_{S}S_{CS}\left(q\right)$$

Therefore, modifying the scattering amplitude of the solvent molecules results in altering the diffraction pattern of the system, such as the appearance or disappearance of peaks as well as modification in relative intensities [25,33]. Note that the use of isotopes does not change the molecular structure [25,32,33].

Neutron diffraction therefore allows one to qualitatively and straightforwardly distinguish a solid solution from a molecular complex thanks to the availability of hydrogenous and deuterated solvents.

### 2.4. Diffraction Data

The diffraction data have all been recorded in a domain of transfer momentum *q* $\left(q=\frac{4\pi }{\lambda }\sin\left(\frac{\theta }{2}\right)\right)$ (*λ* = wavelength, *θ* = scattering angle) ranging from 0.5 to 10 nm^−1^. As the solvent displays a nearly flat scattering pattern in the *q*-range of interest, only the empty cell has been subtracted from the raw spectra. As a result, the slight upturn of the base line at larger *q* is due to the coherent scattering of the solvent. Indeed, the solvent will give a large “bump” peaking at about *q* = 5.7 nm^−1^ arising from its liquid order.

**Figure 3:**
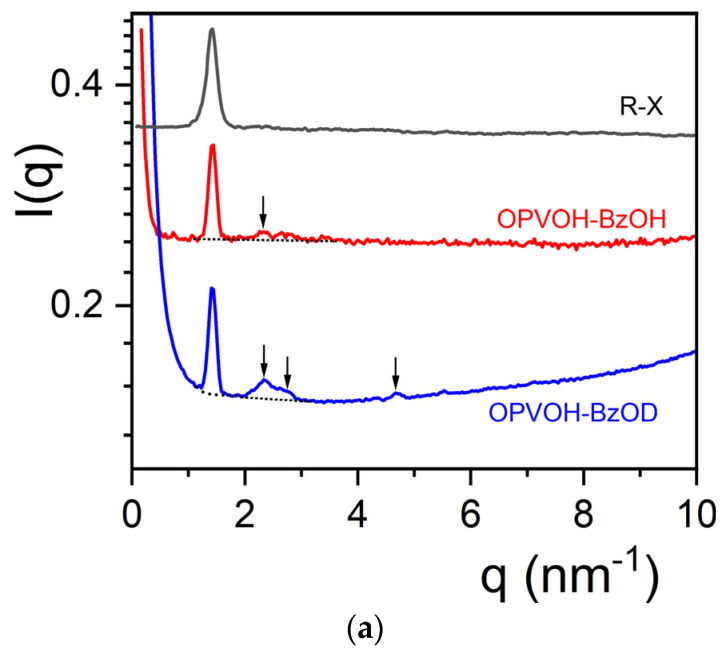

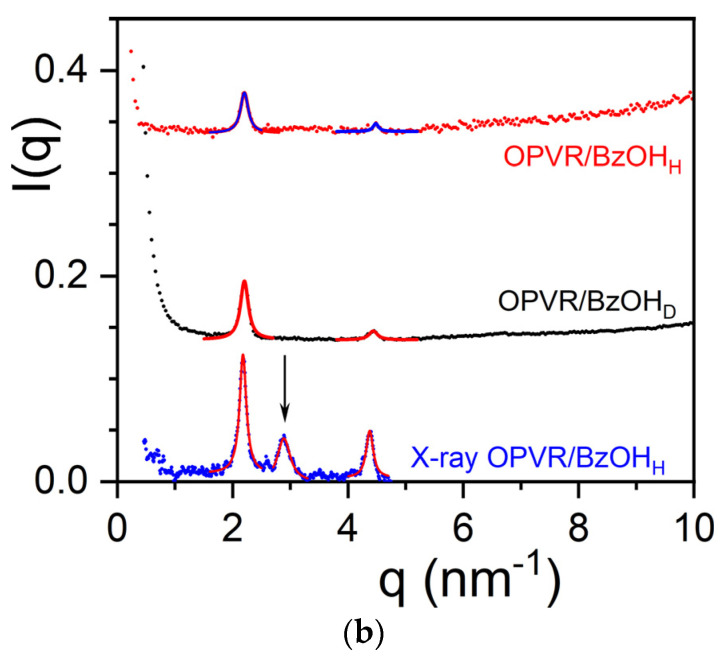
(**a**) Diffraction patterns for OPVOH/benzyl alcohol for C_OPVOH_ = 0.04 g/cm^3^; black line = X-ray for OPVOH/BzOH_H_; red line = neutrons for OPVOH/BzOH_H_; blue line = neutrons for OPVOH/BzOH_D_. (Arrows indicate peaks seen in neutron diffraction.) (**b**) Diffraction patterns for OPVR/benzyl alcohol for C_OPVOH_ = 0.04 g/cm^3^; blue line = X-ray for OPVR/BzOH_H_; red line = OPVR/BzOH_H_; black line = OPVR/BzOH_D_. The arrow indicates the additional peak seen in X-ray diffraction. X-ray data are similar to those reported in ref. [21] by X-ray diffraction from synchrotron radiation. Peaks are fitted with Lorentzian functions for determining the relative intensities (solid lines).

There is a significant difference between the diffraction patterns recorded by X-ray and by neutrons for the OPVOH/benzyl alcohol systems, as seen in Figure 3a. While there is only one peak at *q* = 1.405 nm^−1^ for the X-ray pattern, other peaks appear in the neutron patterns. A weak peak is seen for OPVOH/BzOH_H_ at *q* = 2.329 nm^−1^, compared to three additional peaks for OPVOH/BzOH_D_ at *q* = 2.329 nm^−1^, *q* = 2.79 nm^−1^, and *q* = 4.672 nm^−1^ (see Table 1). Miller indices are assigned to the peaks by taking the π-π stacking direction as the c-axis (Table 1). The relative intensities of the neutron diffraction peaks for each system, *I*(*q_i_*) in %, are calculated by

(7) $$I\left(q_{i}\right)=\sigma_{i}/\sum_{1}^{4}\sigma_{i}$$

where *σ_i_* is the surface of peak *i* as determined by means of a fit with a Lorentzian function:

(8) $$I\left(q\right)=S_{B}+\frac{2A}{\pi }\left[\frac{∆q}{4{\left(q-q_{o}\right)}^{2}+{∆q}^{2}}\right]$$

where *S_B_* is the flat background (solvent + incoherent), *A* is a constant, *q_o_* is the peak maximum, and ∆*q* is the full width at half maximum.

The difference in relative intensities between peaks from OPVOH/BzOH_H_ and OPVOH/BzOH_D_ allows one to conclude unambiguously that OPVOH and benzyl alcohol form a molecular complex (Table 1).

**Figure 4:**
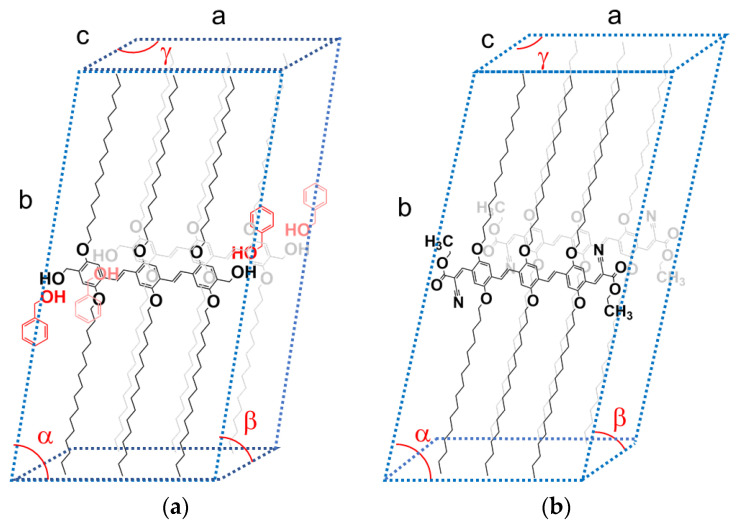
(**a**) Tentative monoclinic crystalline lattice for OPVOH/benzyl alcohol gels with a tentative positioning of the benzyl alcohol molecules; a = 2.7 nm; b = 4.89 nm; c = 0.35 nm; α = 79 ± 2°; β = γ = 90°. (**b**) Tentative triclinic crystalline lattice for OPVR/benzyl alcohol gels based on a previous paper [28]; a = 2.8 nm; b = 4.89 nm; c = 0.49 nm; α = 79 ± 2°; β = 42 ± 2°; γ = 142 ± 4°.

The peak at *q* = 2.329 nm^−1^ for OPVOH/BzOH_D_ corresponds to a distance *d* = 2.7 nm, close to the spacing between the terminal OH groups (≈2.6 nm). It therefore corresponds to the 100 plane, which contains the BzOH molecules. The other peaks are simply second orders of the 010 and 100 planes, which shows the high degree of organization within the OPVOH fibrils. A possible model is drawn in Figure 4a showing how the benzyl alcohol molecule can interact with OPVOH.

Drying OPVOH/benzyl alcohol gel, which results in the removal of the solvent from the crystalline lattice, is likely to introduce some local disorganization. This process may account for the observed collapse of the gel morphology.

The outcomes differ conspicuously in the case of OPVR/benzyl alcohol systems. As seen in Figure 3b, the intensities of the neutron diffraction peaks and their positions are virtually identical whether deuterated or hydrogenous benzyl alcohol is used. This demonstrates the absence of a molecular complex in the case of OPVR.

Interestingly, a third peak is observed by X-ray diffraction which is conspicuously absent in the neutron diffraction patterns. This peak corresponds to the 100 plane containing the C_2_H_5_0 group. The neutron-scattering amplitude of this group is calculated $A_{C2H50}$ as follows:

(9) $$A_{C2H50}=\sum_{Atome}a_{i}$$

where *a_i_* is the scattering amplitude of each atom constituting the group.

Equation (9) gives a value close to $A_{C2H50}=$0 thanks to the negative value of hydrogen (*a_H_* = −0.375) [30]. This therefore accounts for the absence of the 110 peak in the neutron diffraction patterns.

The results are gathered in Table 2 where the peaks are indexed by again taking the π-π stacking direction as the c-axis. A tentative crystalline lattice is displayed in Figure 4b.

## 3. Conclusions

The results presented here provide a conclusive answer to a previous conjecture that predicted the occurrence of a molecular complex of OPVOH molecules in the organogelation process [21]. Conversely, the formation of this complex depends upon the terminal group, as confirmed by the outcomes observed in the case of the OPVR/OPVOH gels. This may have a direct impact on the differing morphologies of these gels (see Figure 2): a hub-like type with thin ribbon-like fibrils in OPVOH/benzyl alcohol systems against a randomly dispersed type in OPVR/benzyl alcohol gels, as well as the collapse of the gel morphology of the latter (Figure 2) [37]. The reason why the formation of an OPVOH/benzyl alcohol complex overcomes the expected OPVOH-OPVOH interaction in the self-assembly process remains unclear so far. Most probably, the OPVOH/benzyl alcohol interaction is already established in the liquid state so that the complex forms straightforwardly upon gelling the solution. The propensity of benzyl alcohol to form dimers through hydrogen bonds is a well-known phenomenon [38]. As a result, a benzyl alcohol molecule may indifferently interact either with the OH terminal groups of OPVOH or another benzyl alcohol molecule.

It is worth emphasizing that the existence of molecular complexes has also been recently demonstrated in the case of triaryl tris amide organogels by means of neutron diffraction [24]. The occurrence of molecular complexes is likely to occur in the gelation process of many other systems, which is something to consider when determining the molecular structure, and in accounting for the thermodynamic properties.

## 4. Materials and Methods

**Materials**: The synthesis and properties of the oligo (p-phenylene vinylene) gelator (OPVOH) are described in reference [17]. The basic chemical structure of the molecule used in this study is shown in Scheme 1. The solvents used for this investigation were benzyl alcohol (BzOH) and benzyl methyl ether (BME). Hydrogenous solvents of high purity grade were purchased from Aldrich and used without further purification. The deuterated benzyl alcohol, C7D7OH, was purchased from Eurisotop (Saclay, France) and used as received. The preparation conditions for the neutron and the X-ray experiments were the same (quenching temperature and ageing).

**Differential Scanning Calorimetry**: Diamond DSC from Perkin Elmer was used to analyze the gel formation and gel melting. Heating and cooling rates ranging from 5 °C/min to 15 °C/min were used. Approximately 30 mg of previously prepared gels was transferred into stainless steel sample pans. These pans were then hermetically sealed by an O-ring to prevent solvent evaporation.

**X-ray Diffraction**: X-ray diffraction experiments were performed by means of a diffractometer developed at the Institut Charles Sadron on the DiffériX platform. The instrument operates with a monochromatic beam of wavelength λ = 0.154 nm and a hybrid photon-counting detector (HPC-Dectrics Pilatus^®^3 R 300K). The sample–detector distance was set so as to access scattering vectors q = 4πsin(θ/2)/λ (θ = diffraction angle) ranging from q = 0.5 to 10 nm^−1^. Calibration of the detector was performed with a silver behenate sample. The gel samples were placed in home-made sealed cells of adjustable thickness.

**Neutron Diffraction**: The neutron diffraction experiments were carried out with D16, a camera located at Institut Laue-Langevin (Grenoble, France) [39]. D16 is equipped with a new curved detector 38 cm high which covers an 86° solid angle with a high angular resolution of 0.075°. This detector is based on the Trench-MultiWire Proportional Chamber (MWPC) detector technology developed at ILL: 6 modules are mounted side by side in a 3He-filled curved vessel [40]. Each module consists of 192 cathode blades positioned every 2 mm, and 192 anode wires spaced by 1.5 mm [41]. The neutron wavelength is selected by beam reflection onto a focussing pyrolytic graphite monochromator. The present experiments were performed with a neutron wavelength of λ = 0.449 nm, giving access to the following transfer momenta range of 0.6 nm^−1^ ≤ q ≤ 10 nm^−1^.

**Optical Microscopy**: Pictures were taken with a NIKON Optiphot-2 equipped with a CCD camera by means of Nomarsky phase contrast. The samples were prepared by re-melting between glass-slides those gels obtained beforehand in a test-tube.

**Scanning Electron Microscopy**: The electron microscopy images were obtained on a JEOL (JSM 6700F) FESEM instrument. Hot solutions of OPV/benzyl alcohol were drop-casted on silicon wafers and subsequently quenched to 0 °C in order to form the gel network. The samples were then dried under vacuum at room temperature. The dried gels were further coated by indium sputtering.

## Figures and Tables

**Table 1:** Positions of the peaks for OPVOH/BzOH_H_ and OPVOH/BzOH_D_ together with the distances calculated by means of Bragg’s law, the corresponding crystallographic planes, and their relative intensities as calculated by Relation (7) (see text for details).

Peak Position (nm^−1^)	*d_hkl_* (nm)	*hkl*	Relative Intensity I(q_i_) in (%) BzOH_D_	Relative Intensity I(q_i_) in (%) BzOH_H_
1.405 (also X-ray)	4.472	010	64	95
2.329	2.698	100	28	5
2.790	2.252	020	3	-
4.672	1.345	200	5	-

**Table 2:** Positions of the X-ray and neutron diffraction peaks for OPVR/BzOH together with the distances calculated by means of Bragg’s law, and the corresponding crystallographic planes.

X-Ray Peak (nm^−1^)	Neutron Peak (nm^−1^)	*d_hkl_* (nm)	*hkl*
2.19	2.20	2.87 ± 0.1	010
2.894	/	2.17	100
4.4	4.44	1.42 ± 0.5	020

## Data Availability

The raw data supporting the conclusions of this article will be made available by the authors on request.
